# A Comparison of Measurement Methods and Sexual Dimorphism for Digit Ratio (2D:4D) in Han Ethnicity

**DOI:** 10.1007/s10508-013-0179-9

**Published:** 2013-09-07

**Authors:** Huanjiu Xi, Ming Li, Yingnan Fan, Liguang Zhao

**Affiliations:** 1 Institute of Anthropology, Liaoning Medical University, Jinzhou, 121001 People’s Republic of China; 2 Chongqing Police Bureau, Chongqing, People’s Republic of China

**Keywords:** 2D:4D, Digit ratio, Sex differences, Ethnicity

## Abstract

The digit ratio (2D:4D) is sexually dimorphic and has been considered an indicator of prenatal sex hormone exposure. Previous studies have shown that males tend to have lower 2D to 4D ratio than females, and this sexual dimorphism has been reported across different ethnic groups and different countries. However, digit ratio data are missing from the Han ethnicity in China. Furthermore, most of the previous studies used direct measurement for digit ratio. In this article, we used multiple measurement methods, including the direct measurement and two X-ray measurement methods to examine the trait of 2D:4D in Chinese Han. Our sample consisted of 128 men and 122 women from Liaoning Medical University. They were 18–20 years old. The direct measurement and two types of X-ray measurements of the length of their 2nd and 4th fingers were used separately to calculate digit ratios. Soft tissue thickness of 2D and 4D fingertips were also assessed from the two X-ray methods. The results suggest that (1) sex differences in 2D:4D tend to be stronger in the two X-ray measurements in comparison to the direct measurement; (2) 2D:4D ratios from X-ray measurements tend to be lower than that from the direct measurement; (3) Han ethnicity have a lower mean value of 2D:4D than other ethnic groups; (4) no sex difference in the soft tissue of finger tips. In conclusion, the digit ratio is lower in both men and women in Han, and the sexual dimorphism in digit ratio was stronger with X-ray measurements in comparison to the direct measurement.

## Introduction

Digit ratio (2D:4D) is defined as the ratio of the index finger and ring finger length. Manning, Scutt, Wilson, and Lewis-Jones (1998) first reported a sexual dimorphism in digit ratio: on average, men have a lower ratio than women. Subsequently, it has been suggested that 2D:4D may be a possible predictor for many diseases that are correlated with sex-linked traits. Hence, the digit ratio may be a useful risk indicator for identifying people with high susceptibility to certain diseases so interventions can be started earlier to prevent the occurrence of these diseases (Manning & Bundred, 2000; Ozdogmus et al., 2010). By 2009, there were more than 300 publications about the digit ratio (Voracek & Loibl, 2009). The digit ratio has been proposed as a putative marker of prenatal hormone exposure (Schwerdtfeger, Heims, & Heer, 2010). However, no digit ratio study has been conducted in Chinese populations.

The measurement methods of 2D:4D include direct measurement, photocopy, self-report online, and X-ray measurement. Most of the early studies employed the direct measurement method (e.g., Manning & Taylor, 2001; Manning et al., 1998). The direct measurement shows a high degree of repeatability (Manning, 1995; Scutt & Manning, 1996). Comparison studies of 2D:4D measurement methods have been done (Allaway, Bloski, Pierson, & Lujan, 2009; Caswell & Manning, 2009; Kemper & Schwerdtfeger, 2009; Manning, Fink, Neave, & Caswell, 2005). Direct measurement has been used widely and is thought to be the most accurate and fastest method. However, the direct measurement is difficult to conduct when the participants moved frequently and particularly so in studies of children. In comparison to the direct measurement method, radiographic measurements are much easier to make and can be retained as a permanent record. Using radiographic measurement, Vehmas, Solovieva, and Leino-Arjas (2006) proposed that soft tissues of fingers rather than the length of finger bones may be related to the 2D:4D ratio.

The objective of this study was to compare the application of the direct measurement method with the applications of the two X-ray measurements methods in measuring the digit ratio among men and women from the Han ethnicity in Northern China and to examine whether the soft tissues at the finger tips were the significant contributors to sex difference in digit ratio.

## Method

### Participants

A total of 250 healthy undergraduate students (128 males, 122 females) aged 18–20 years old were recruited from Liaoning Medical University in China. People with a history of injury or illness affecting two hands or fingers were excluded. Because of ethnicity and latitude variations in the digit ratio, participants were included in this study only if they had always lived in Liaoning and belonged to the Han ethnicity. Informed consent was received from all the participants. Ethical approval was granted by the local Ethics Committees.

### Measures

**Fig. 1:**
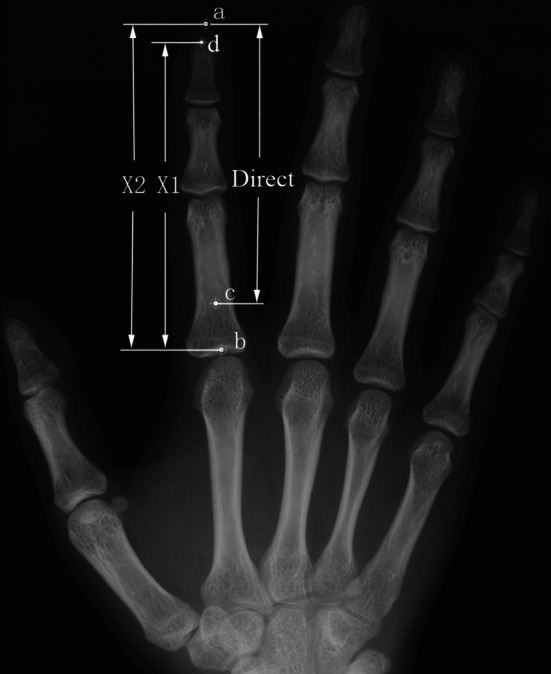
The methods of measurement: (1) direct measurement (*c*)–(*a*); (2) X1-bases of 2nd/4th proximal phalanx to tip of corresponding distal phalanx (*b*)–(*d*); (3) X2-bases of 2nd/4th proximal phalanx to distal of soft tissue (*b*)–(*a*)

Participants were asked to remove any jewelry or rings that would interfere with finger length measurements. X-rays were taken from the dorsal surface of two hands at 70 kvp, 10 mA, and 1 s at a collimator distance of 70 cm using Siemens analogue equipment. Radiographs were inspected at lighted view boxes, first using the method (X1) by Vehmas et al. (2006). This method measured the length from the bases of the second or fourth proximal phalanxes to the tips of the corresponding distal phalanxes. The length of the fingers was also measured using a new method (X2) developed in this study. In this method, finger length was measured from the bases proximal phalanxes to the distal of the soft tissue (Fig. 1). The soft tissue thickness of fingertip was calculated as the difference between X2 and X1. Following Manning’s method, the direct measurement was measured from the basal crease of the finger proximal to the palm of the hand to the tip of the finger.

The length of 2D and 4D was measured with a vernier caliper which recorded to 0.02 mm. All the observers (research staff) received training on both direct and X-ray finger measurements and the reliability of these measurements were assessed. The correlation coefficients of the inter-observers/intra-observers were 0.87–0.96. Lengths of 2D and 4D of two hands were measured by two observers using three different methods.

## Results

### Radio of 2nd and 4th Digit Length

Table 1 shows the means, SDs, and sex differences for 2D:4D ratios and Table 2 shows the means and SDs of the length of 2D and 4D from three different measurement methods. A significant sexual dimorphism in 2D:4D ratio was observed in all measurements from the three methods. Digit ratios appeared lower in men than in women, and sex differences were significant on the right hand, but not the left hand, in the direct measurement method (*p* < .01). Result from the two X-ray measurement methods showed that 2D:4D ratio had significant sex differences on both hands and similar to the above results the digit ratio was lower in men than in women (*p* < .01). Second finger length tended to be lower than 4D regardless of the method. In comparison to measurements of finger length from the direct method, the length for 2D and 4D on both hands from X-ray measurement methods was obviously longer and the span of 2D and 4D was greater.

**Table 1:** Measurements and sexual differences of 2D:4D ratio by three methods

Method	Left hand				Right hand			
	*M*	SD	*t*	*p*	*M*	SD	*t*	*p*
Direct								
Men	0.96	0.03	−1.21	ns	0.95	0.03	−3.27	<.001
Women	0.97	0.03			0.96	0.03		
X1								
Men	0.92	0.02	−4.56	<.001	0.92	0.02	−5.43	<.001
Women	0.93	0.02			0.93	0.02		
X2								
Men	0.92	0.02	−4.41	<.001	0.92	0.02	−3.99	<.001
Women	0.93	0.02			0.93	0.02

**Table 2:** Mean (in cm) and SD of length for 2D and 4D by three methods

Method	Left hand				Right hand			
	2D		4D		2D		4D	
	*M*	SD	*M*	SD	*M*	SD	*M*	SD
Direct								
Men	7.14	0.40	7.41	0.45	7.08	0.40	7.43	0.44
Women	6.69	0.44	6.91	0.45	6.66	0.42	6.91	0.42
X1								
Men	8.19	0.49	8.90	0.55	8.17	0.48	8.93	0.55
Women	7.60	0.47	8.16	0.50	7.61	0.47	8.20	0.49
X2								
Men	8.48	0.56	9.25	0.63	8.48	0.55	9.24	0.63
Women	7.89	0.54	8.50	0.57	7.91	0.53	8.53	0.56

### Differences among Methods

Direct measurements significantly differed from the measurements from the two types of X-ray methods (*p* < .01) while the differences between the two X-ray methods, X1 and X2, were not statistically significant for 2D and 4D length. X1 and X2 yielded lower digit ratios than direct finger measurement. The means of 2D:4D ratio on the hands in both men and women were the largest in the direct measurement. The soft tissue thickness appeared to be slightly higher in men than in women (except on the right hand in women), but the sex difference did not reach statistical significance (Table 3).

**Table 3:** Soft tissue thickness (in cm) at fingertip measured by X-ray

	Left hand								Right hand							
	2D				4D				2D				4D			
	*M*	SD	*t*	*p*	*M*	SD	*t*	*p*	*M*	SD	*t*	*p*	*M*	SD	*t*	*p*
Male	0.30	0.22	<1	ns	0.35	0.24	<1	ns	0.31	0.23	<1	ns	0.31	0.25	<1	ns
Female	0.29	0.21			0.33	0.23			0.31	0.23			0.33	0.25		

## Discussion

Many studies have suggested that 2D:4D ratio is correlated with the level of sex hormones in utero (Brown, Hines, Fane, & Breedlove, 2002; Ciumas, Linden Hirschberg, & Savic, 2009; Okten, Kalyoncu, & Yaris, 2002) and 2D:4D has significant sex and ethnic differences (Manning, Stewart, Bundred, & Trivers, 2004; Manning et al., 2000a). But the majority of the results were based on direct measurement and the result of 2D:4D from X-ray measurement in relation to Han ethnicity in China was unknown. Previous studies have shown that men had lower 2D:4D than women and the sex differences were greater for the right hand 2D:4D than for the left hand (Grimbos, Dawood, Burriss, Zucker, & Puts, 2010; Manning et al., 1998).

In this study, we found that the results of three measurement methods were not completely in agreement (Table 1). In the direct measurement, only the right hand showed significant sex differences while there was no significant sex difference on the left hand, which is similar to the results in Manning et al. (1998) (see also Reuter & McQuade, 2009). However, sexual dimorphism in 2D:4D ratios were observed on both hands when X-ray measurement methods were used. This result from our study was different from two other studies with X-ray measurements (Manning, Trivers, Thornhill, & Singh, 2000b; McIntyre, Ellison, Lieberman, Demerath, & Towne, 2005). Although the results were not completely uniform regarding the hands, our results still support significant sexual dimorphism in 2D:4D as reported from previous studies, regardless of the methods used.

It has been suggested that mean 2D:4D ratio differs across ethnic groups in both men and women, with a higher 2D:4D ratio in Caucasians and a lower 2D:4D ratio in Blacks and Chinese. However, in one study, the mean 2D:4D of Chinese was 0.974 and 0.971 on the left and right hand in men, and 0.986 and 0.982 in women (Manning, Churchill, & Peters, 2007), which are different from the mean values from our study. There are 56 ethnic groups in different latitude regions in China and the selection of different ethnic groups or regions in each study was likely to be the reason for the observed difference in studies of Chinese. Nevertheless, the results from our study were in agreement with the previous studies showing lower 2D:4D ratios in the Han ethnicity when the direct measurement method was used.

One Finnish study using the X-ray (X1) method reported the mean 2D:4D was 0.925 on the right hand and 0.924 on the left or both hands. In comparison to the Finnish study, our results showed higher digit ratios in Chinese women than in the Finnish women (Manning et al., 2004).

Significant differences in digit ratios by measurement method were observed in our study, suggesting variations in study findings from previous published research may be due to using different measurement methods.

The length of 2D and 4D was measured from the basal crease of the finger proximal to the palm of the hand to the tip of the finger in the direct measurement (Caswell & Manning, 2009; Manning et al., 2005), which was different from X-ray measurements taken from the bases of the second and fourth proximal phalanges to the tips of the corresponding distal phalanges or the distal of the soft tissue. From the point of anatomical location view, the place of proximal phalanx in X-ray measurement was below basal crease that is used in the direct measurement. Compared with the direct measurement, the length of 2D and 4D in X-ray measurement method was longer and the 2D:4D ratio was smaller. Vehmas et al. (2006) applied the X1 method to study the associations of the 2D:4D index with various sex-linked features and suggested that differences in 2D:4D ratio may be due to differences in the soft tissues of fingers rather than to the length of bones.

To explore the role of the soft tissue of fingertip for digit ratio, we used two X-ray methods, and calculated the soft tissue thickness of each fingertip, and examined correlations between the soft tissue thickness and 2D:4D ratio. Our results showed no statistically significant sex differences in 2D:4D finger soft tissue thickness and no significant correlations between 2nd or 4th finger soft tissue thickness and 2D:4D ratio, except the second finger soft tissue thickness (men right and women left) from X1 measurement (*p* < .05; *r* = .20–.24). Thus, the soft tissues at the fingertips were not the significant contributors to the sex difference in digit ratio. Given the findings in our study, X-ray measurement methods are preferred to explore whether the basal crease or the bone length is affected by prenatal hormone exposure.

In conclusion, results from this study have indicated that X-ray may be the method to be used in future studies on digit ratios. In addition, this study has confirmed previous findings of sexual dimorphism in 2D:4D ratio and added data on mean digit ratios in the Chinese Han ethnicity. The soft tissues at the finger tips were not significant contributors to the sex difference in digit ratio.

## References

[CR1] Allaway HC, Bloski TJ, Pierson RA, Lujan ME (2009). Digit ratios (2D:4D) determined by computer-assisted analysis are more reliable than those using physical measurements, photocopies and printed scans. American Journal of Human Biology.

[CR2] Brown WM, Hines M, Fane BA, Breedlove SM (2002). Masculinized finger length patterns in human males and females with congenital adrenal hyperplasia. Hormones and Behavior.

[CR3] Caswell N, Manning JT (2009). A comparison of finger 2D:4D by self-report direct measurement and experimenter measurement from photocopy: Methodological issues. Archives of Sexual Behavior.

[CR4] Ciumas C, Linden Hirschberg A, Savic I (2009). High fetal testosterone and sexually dimorphic cerebral networks in females. Cerebral Cortex.

[CR5] Grimbos T, Dawood K, Burriss RP, Zucker KJ, Puts DA (2010). Sexual orientation and the second to fourth finger length ratio: A meta-analysis in men and women. Behavioral Neuroscience.

[CR6] Kemper CJ, Schwerdtfeger A (2009). Comparing indirect methods of digit ratio (2D:4D) measurement. American Journal of Human Biology.

[CR7] Manning JT (1995). Fluctuating asymmetry and bodyweight in men and women: Implications for sexual selection. Ethology and Sociobiology.

[CR8] Manning, J. T., Barley, L., Walton, J., Lewis-Jones, D. I., Trivers, R. L., Singh, D., … Szwed, A. (2000a). The 2nd:4th digit ratio, sexual dimorphism, population differences, and reproductive success: Evidence for sexually antagonistic genes? *Evolution and Human Behavior, 21*, 163–183. 10.1016/s1090-5138(00)00029-5 10828555

[CR9] Manning JT, Bundred PE (2000). The ratio of 2nd to 4th digit length: A new predictor of disease predisposition?. Medical Hypotheses.

[CR10] Manning JT, Churchill AJ, Peters M (2007). The effects of sex, ethnicity, and sexual orientation on self-measured digit ratio (2D:4D). Archives of Sexual Behavior.

[CR11] Manning JT, Fink B, Neave N, Caswell N (2005). Photocopies yield lower digit ratios (2D:4D) than direct finger measurements. Archives of Sexual Behavior.

[CR12] Manning JT, Scutt D, Wilson J, Lewis-Jones DI (1998). The ratio of 2nd to 4th digit length: A predictor of sperm numbers and concentrations of testosterone, luteinizing hormone and oestrogen. Human Reproduction.

[CR13] Manning JT, Stewart A, Bundred PE, Trivers RL (2004). Sex and ethnic differences in 2nd to 4th digit ratio of children. Early Human Development.

[CR14] Manning JT, Taylor RP (2001). Second to fourth digit ratio and male ability in sport: Implications for sexual selection in humans. Evolution and Human Behavior.

[CR15] Manning JT, Trivers RL, Thornhill R, Singh D (2000). The 2nd:4th digit ratio and asymmetry of hand performance in Jamaican children. Laterality.

[CR16] McIntyre MH, Ellison PT, Lieberman DE, Demerath E, Towne B (2005). The development of sex differences in digital formula from infancy in the Fels Longitudinal Study. Proceedings: Biological Sciences.

[CR17] Okten A, Kalyoncu M, Yaris N (2002). The ratio of second-and fourth-digit lengths and congenital adrenal hyperplasia due to 21-hydroxylase deficiency. Early Human Development.

[CR18] Ozdogmus O, Cakmak YO, Coskun M, Verimli U, Cavdar S, Uzun I (2010). The high 2D:4D finger length ratio effects on atherosclerotic plaque development. Atherosclerosis.

[CR19] Reuter C, McQuade DB (2009). Sex and hand differences in circadian wrist activity are independent from sex and hand differences in 2D:4D. Journal of Circadian Rhythms.

[CR20] Schwerdtfeger A, Heims R, Heer J (2010). Digit ratio (2D:4D) is associated with traffic violations for male frequent car drivers. Accident Analysis and Prevention.

[CR21] Scutt D, Manning JT (1996). Symmetry and ovulation in women. Human Reproduction.

[CR22] Vehmas T, Solovieva S, Leino-Arjas P (2006). Radiographic 2D:4D index in females: No relation to anthropometric, behavioral, nutritional, health-related, occupational or fertility variables. Journal of Negative Results in BioMedicine.

[CR23] Voracek M, Loibl LM (2009). Scientometric analysis and bibliography of digit ratio (2D:4D) research, 1998–2008. Psychological Reports.

